# The Economic impact of Non-communicable Diseases on households in India

**DOI:** 10.1186/1744-8603-8-9

**Published:** 2012-04-25

**Authors:** Michael M Engelgau, Anup Karan, Ajay Mahal

**Affiliations:** 1 National Center for Chronic Disease Prevention and Health Promotion, Centers for Disease Control and Prevention, Atlanta, Georgia, USA; 2 Department of Public Health, University of Oxford, Oxford, UK; 3 Public Health Foundation of India, New Delhi, India; 4 School of Public Health and Preventive Medicine, Monash University, 99 Commercial Road, Melbourne 3004, VIC, Australia

## Abstract

**Background:**

In India, Non Communicable Diseases (NCDs) and injuries account for an estimated 62% of the total age-standardized burden of forgone Disability Adjusted Life Years (DALYs). Public and private financing of clinical services to reduce the NCD burden is a major challenge.

**Methods:**

We used National Sample Survey Organization (NSSO) survey data from 1995-96 and 2004 covering nearly 200 thousand households to assess healthcare utilization patterns and out of pocket health spending by disease category. For this purpose, self-reported diseases and conditions were categorized into NCDs and non-NCDs. Survey data were used to assess how households financed their overall health expenditures and related this pattern to specific health conditions. We measured catastrophic spending on NCD-related hospitalization, defined as occurring when health expenditures exceeded 40% of a household's ability to pay, that is, household consumption spending *less *combined survival consumption expenditure; and impoverishment when per capita expenditure within the household decreased to below the poverty line once health spending was netted out.

**Results:**

The share of NCDs in out of pocket health expenses incurred by households increased over time, from 31.6 percent in 1995-96 to 47.3 percent in 2004. In both years, own savings and income were the most important source of financing for many health conditions, typically between 40-60 percent of all spending, whereas 30-35 percent was from borrowing. The odds of catastrophic hospitalization expenditures for cancer was nearly 170% greater and for CVD and injuries 22 percent greater than the odds due to communicable diseases. Impoverishment patterns were similar.

**Conclusions:**

Out of pocket expenses for treating NCDs rose sharply over the period from 1995-96 to 2004. When NCDs are present, the financial risks to which Indians households are exposed are significant.

## Background

Non-communicable diseases (NCDs), primarily chronic diseases (heart disease, diabetes, cancer, and chronic respiratory disease/asthma) and injuries, and mental illness, now account for an estimated 62% of the total age-standardized burden of forgone disability adjusted life-years (DALYs) in India, with the remainder from communicable diseases and maternal and child health issues [1]. Risk factors such as tobacco use and a growing morbidity from obesity, heart disease, diabetes, cancer, and chronic respiratory disease along with injuries account for this share. A recent study of adult mortality estimated that 1 in 5 deaths among men and 1 in 20 deaths among women in India were due to tobacco smoking, and the most recent Indian National Family Health Survey found overweight or obesity among 13% to 25% of men and 19% to 39% of women [2,3]. In addition, transportation gains and new and faster roads can lead to more injuries [4,5]. Finally a legacy of under-nutrition during fetal development and early childhood and its association with NCDs later in life also adds to this risk burden [6-8].

Developed countries have experienced substantial heart disease burden reductions over the last several decades, and recent studies have found that both population-level risk factor reduction and clinic-base primary care treatments for those already affected, are equally responsible for these declines [1,9-12]. Thus, for developing countries such as India, along with prevention efforts, primary care treatments are an important element for reducing the NCD burden. Delivering this care in developing countries has many challenges, however. Even when such care is available, individuals with NCDs will continue to face significant risks of hospitalization and the associated costs of financing care. While financing is important for all diseases and health conditions, our focus in this study is on NCDs because of their current large burden.

Social and private health insurance that can help finance health services are currently limited in India and until recently, government financing efforts were mostly focused on subsidizing public facilities. In this context, large household expenses for care of a member can consume a substantial proportion of the household's income, especially for NCDs that are likely to be expensive to treat. To better understand the associated burden on households, we estimate annual household expenses for NCD care and how they are paid for, including out of pocket (e.g. from household income and saving), support from (non household) family and friends, and other means such as selling assets. We then explore the implications of these household spending patterns since high levels of out of pocket payments increase the risk of catastrophic spending [13] and can also lead to impoverishment [14].

## Methods

### Data Sources

We used data from household health surveys conducted by the National Sample Survey Organization (NSSO) of India in 1995-96 and 2004 to assess healthcare utilization patterns and out of pocket health spending. The 1995-96 survey covered nearly 120,000 households and some 600,000 individuals; the 2004 survey covered nearly 80,000 households and some 380,000 individuals. Since both are stratified random sample surveys, all our estimates are derived by applying sampling weights supplied by the NSSO.

### Disease and Condition Classification

**Figure 1:**
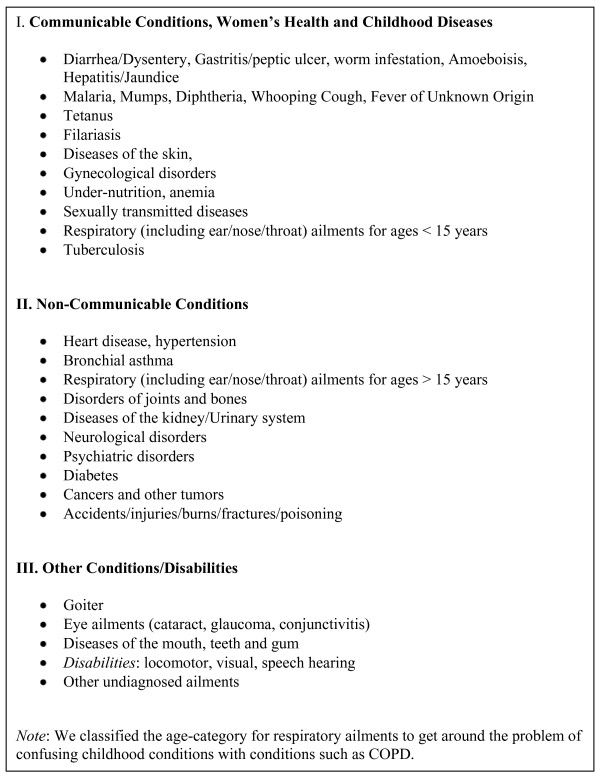
**Classification of 2004 Household Survey response categories from National Sample Survey Data into disease categories**.

Health conditions provided for in the household health care utilization and expenditure surveys were self-reported. We matched the categories in the surveys to broad ICD-9 disease classification to distinguish between major NCD categories (including injuries) and communicable diseases (Figure 1). Some of the disease categories in the surveys could potentially include both NCDs and non-NCDs, so we focused most of our analyses on categories that were clearly NCDs. For example, for the category "respiratory conditions, including ear/nose/throat ailments", the two conditions available were asthma and tuberculosis, and we focused on the former. For cardiovascular disease we focused on the two explicit categories provided: heart disease and hypertension. About 10% of the health conditions could not be identified by the respondents in the survey and thus not classified.

### Hospital Stay and Outpatient Visits

Participants were asked about all hospital stays during the year previous to the survey. For outpatient visits, an initial query about ailments during the 15 days prior to the survey was followed by a question on the healthcare provider visited. The focus on a single provider in the survey is likely to result in underestimates for outpatient visits and expenditures, although recent studies suggest that this error is likely to be small [15]. To estimate the annual number of outpatient visits for the population, we multiplied the number of visits reported in the 15-day reference period by 24.33 (= 365/15). The use of this multiplier is not appropriate for estimating the annual number of visits for a specific household, since that requires the assumption that outpatient visits for that household are distributed uniformly across 15-day periods throughout the year. However, this assumption is less of a concern for calculating aggregate outpatient care use for the full set of households, given that the households and the corresponding 15-day reference period are randomly chosen.

### Financing of Health Services for NCDs

We estimated how households financed their overall health expenses and related this pattern to specific health conditions. Since only household level information on funding sources was available in the 2004 survey, we assumed that the description of the way households financed their health spending corresponded most closely to the health condition on which most household money was spent. By contrast, information on the sources of financing for out of pocket spending in the 1995-96 survey was collected at the individual level by episode of illness, lending itself to more straightforward calculation.

We also analyzed how the burden of household health financing in general (and specifically expenses on NCDs) differed across different socioeconomic groups. For this purpose, we divided the population into quintiles ranked by per capita household expenditures and assessed how health spending varied across quintiles for rural and urban populations.

### Catastrophic Spending and Impoverishment among Households

We measured catastrophic spending on NCD-related hospitalization, which was defined as occurring when health expenses on hospitalization for a given household exceeded 40% of the *ability to pay *[13]: household consumption spending *less *combined survival income for all household members based on poverty line estimates for different states and regions of India. We also measured whether health spending would be impoverishing. Specifically, we considered total hospitalization spending as impoverishing if, after subtracting it from total household spending, a household's expenditure on other items fell below the poverty line level of expenditure (Additional File 1). Given the chronic nature of most NCDs, frequent out of pocket spending on outpatient care can lead to a household becoming impoverished even in the absence of hospitalization. However, impoverishment associated with outpatient care was impossible to assess from our survey data because the relevant information was available only for a 15-day window for a given household. Underestimation of impacts on catastrophic spending and impoverishment could also occur due to underreporting of outpatient treatment for chronic conditions.

## Results

### Utilization of services

Hospital stays overall and for NCDs substantially increased between 1995-96 and 2004. In 2004 there were approximately 30.6 million hospital stays in India, double that in 1995-96 (15.2 million). The proportion of hospital stays due to NCDs increased from 32% (4.8 million stays in total) in 1995-96 to 40% (12.2 million stays in total) in 2004. Of the major NCDs, injuries were the most common reason for a hospital stay, followed by heart disease, cancer, hypertension, and diabetes (Table 1). Hospital stays for all NCDs more than doubled during the study timeframe. The increase (in percentage terms) was greatest for diabetes (278%) followed by injuries (172%), asthma (164%) heart disease (127%), hypertension (126%) and cancer (103%).

**Table 1:** Hospital Stays and Outpatient Visits for Major NCDs in India, 1995-96 and 2004

**Disease Category**	**Hospital Stays** **(millions)**		**Outpatient Visits** **(millions)**	
	
	**1995-96**	**2004**	**1995-96**	**2004**
***All Cause***	*15.21*	*30.58*	*1,294.23*	*2,529.73*
***All NCD***	*4.76*	*12.20*	*279.51*	*893.68*
**Heart Disease**	0.73	1.66	18.85	67.60
**Hypertension**	0.30	0.69	26.19	129.85
**Diabetes**	0.16	0.60	18.20	88.83
**Cancer**	0.44	0.90	6.07	13.37
**Asthma**	0.38	1.01	84.31	97.64
**Injuries**	1.09	2.96	26.66	67.22

*Source*: Authors' estimates using National Sample Survey data for 1995-96 and 2004

The proportion of all hospital stays in the public sector declined to 41% in 2004 from 44% in 1995-96. In 2004, within the major NCD categories the share of the public sector hospital stays was highest for cancers (49%), followed by asthma (45%), injuries (44%), heart disease (39%), and was lowest for diabetes (32%) and hypertension (30%), **(**Table 2).

**Table 2:** Share of Public Sector in Hospital Stays and Outpatient Visits for Major NCDs in India, 1995-96 and 2004

**Disease Category**	**Hospital Stays** **(percent of total)**		**Outpatient Visits** **(percent of total)**	
	
	**1995-96**	**2004**	**1995-96**	**2004**
***All Cause***	*44.0*	*40.7*	*14.5*	*18.0*
***All NCD***	*44.0*	*39.7*	*14.0*	*19.7*
**Heart Disease**	37.8	39.2	17.9	26.0
**Hypertension**	35.7	29.5	12.8	19.5
**Diabetes**	38.5	32.0	18.4	18.2
**Cancer**	52.7	48.7	24.2	31.6
**Asthma**	40.7	45.0	13.4	21.9
**Injuries**	52.1	44.3	17.6	23.5

*Source*: Authors' estimates using National Sample Survey data for 1995-96 and 2004

In 2004, approximately 2.5 billion visits occurred, up from 1.3 billion in the earlier timeframe. The proportion for NCDs increased from 22% (280 million) to 35% (894 million). The increase in the number of visits occurred across the different NCDs, but with wide variations. Outpatient visits for asthma increased the least while hypertension and diabetes had the largest increases (400% and 389%, respectively) (Table 1). Cancer visits, in spite of a doubling in the number of visits, remained the least common. The share of private sector providers in outpatient visits remained high in both time periods, at more than 80% (Table 2).

### Out of Pocket Expenses

Overall, out of pocket expenses increased during the period from 1995-96 and 2004. Nearly 846 billion Indian rupees (INR) were spent out of pocket on health care expenses in 2004, amounting to 3.3% of that year's gross domestic product (GDP). This marked a substantial increase from INR 315 billion (in current INR) spent out of pocket on health care in 1995-96 (about 2.9% of the GDP). The share of NCDs in aggregate household out of pocket health expenses also increased over time, from 31.6% in 1995-96 to 47.3% in 2004, indicating the growing importance of NCDs in terms of their financial impact on households.

**Figure 2:**
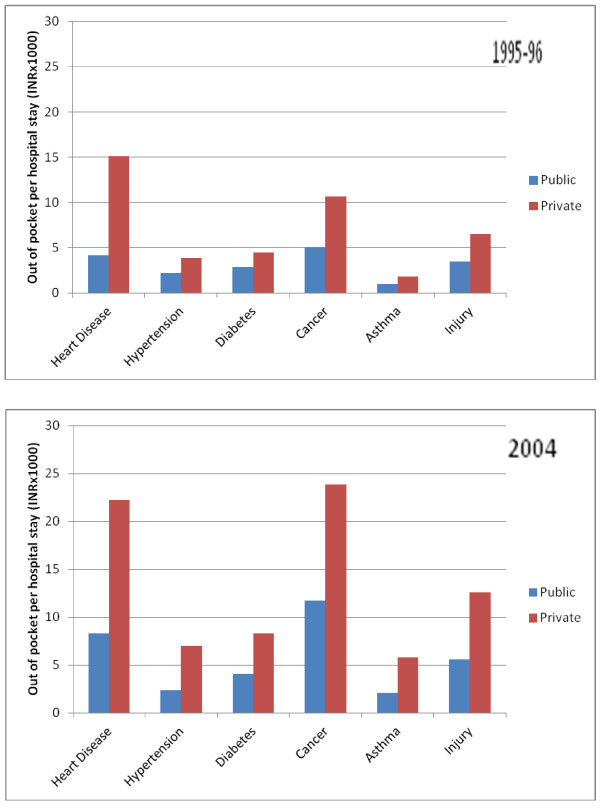
**Out of pocket expenses per hospital stay in public and private systems among those with major NCDs in India, 1995-96 and 2004**.

**Figure 3:**
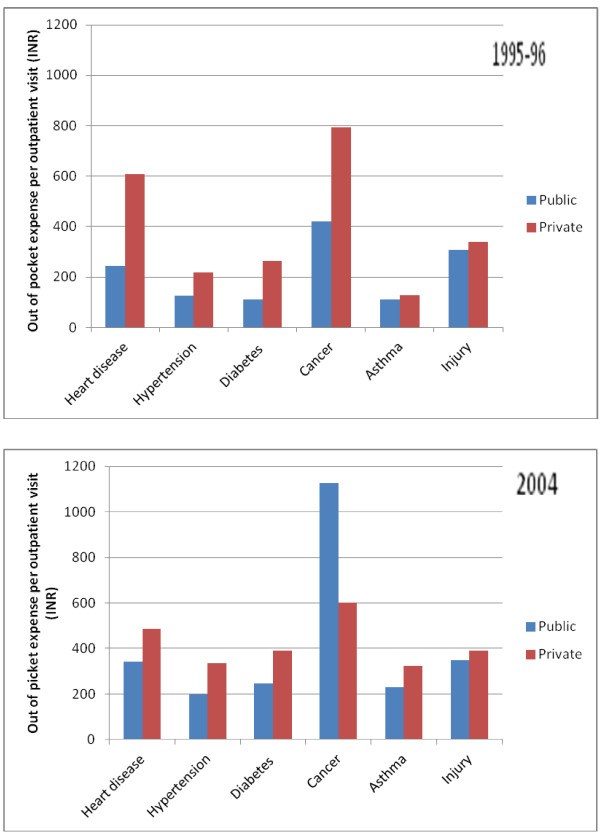
**Out of pocket expenses per outpatient visit in public and private systems among those with major NCDs in India, 1995-96 and 2004**.

Within the major NCD categories, out of pocket expenses per hospital stay and per outpatient visit were particularly high for cancer, heart disease, and injury (Figure 2 and 3). Roughly half of the out-of-pocket expenses on health care were incurred on purchases of medicines, diagnostic tests and medical appliances. A major portion of overall out of pocket health spending (in excess of 45 percent) was for medicines for NCDs and this proportion was as high as 64% and 58% for cases of hypertension and diabetes, respectively. Consultation fees accounted for 5% to 12% of total out of pocket expenses depending on the health condition.

### Paying for Care and Household Financial Vulnerability to NCD

**Figure 4:**
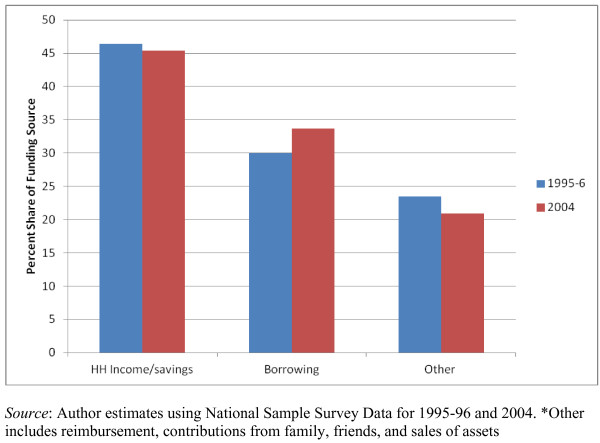
**Source of funds for out of pocket spending on health care for hospital stays in India, 1995-96 and 2004**.

The major source of financing for NCD care was household savings and income, accounting for about 45% of NCD-related out of pocket expenses, with a range of 40% to 60% across the different NCDs (Figure 4). Borrowing accounted for 30% to 35% percent of NCD expenses out of pocket. The terms of borrowing, that is, with (or without) a collateral and the interest are not available in our data. In 2004, about 10% to 15% of expenses were provided by friends and family, a form of community insurance. In both periods, approximately 5% to 6% percent of out of pocket spending on NCD-related hospital stays was reimbursed by employer and insurance companies. In 2004, some of the more expensive to treat health conditions (CVD, cancers, accidents and injuries) involved larger shares of financing from asset sales (included in the "other" category in Figure 4).

**Figure 5:**
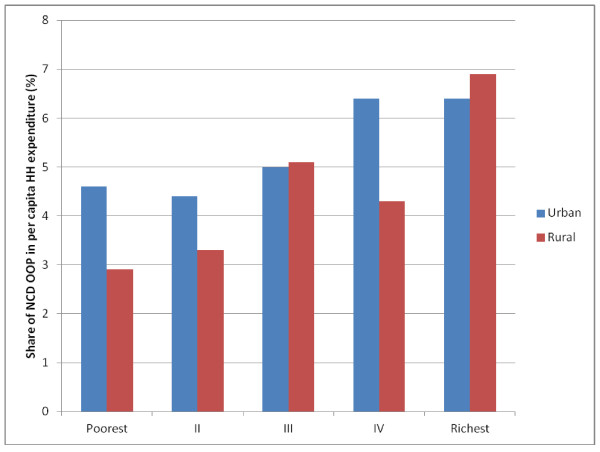
**Share of per capita household income spent on out of pocket expenses for healthcare by expenditure quintiles in urban and rural area in India, 2004**.

Out of pocket health spending, taken as a proportion of household expenditure, did not vary much across household ranking by expenditure quintiles, whether for the population as a whole, or separately by rural and urban populations and fluctuated between 10% to 12% of mean per capita household expenditure. However, we observed that the share of out of pocket expenditures for NCD in total household expenditure rose from poorest to the richest groups. We note also that urban populations allocated a greater share of their out of pocket health expenses on NCDs, compared to their rural counterparts (Figure 5). Although these findings appear to go against the idea that NCDs are creating a financial burden on the poor, the more plausible scenario is that this group seeks less care for these conditions, with potentially adverse implications for employment and incomes [16]. Moreover, because individuals belonging to the lowest expenditure quintile live much closer to the survival threshold, allocating even smaller proportions of income is likely to increase their likelihood to falling below the poverty line. Elsewhere, it has been shown, using the same survey data that we use, that conditional on reporting an ailment, the poorest groups are much less likely to seek treatment than their richer counterparts [17]

### Catastrophic Expenditures and Medical Impoverishment for NCDs

Table 3 presents results from a logit regression of indicators for household catastrophic spending and impoverishment for NCDs and communicable disease among individuals who experienced hospital stays in 2004. Our results show that the odds of catastrophic spending and impoverishment are higher for those hospitalized with NCDs than for those hospitalized with communicable conditions. Hospitalization with CVD resulted in 12% higher odds of incurring catastrophic spending and 37% greater odds of falling into poverty. For cancer, the impact was greatest with the odds of catastrophic expenditures 170% higher than the odds of incurring catastrophic spending when hospital stays are due to a communicable condition. A 133% likelihood of falling into poverty was found. These basic findings persisted even when we disaggregated the analysis by household expenditure quintiles, ranging from the poorest to the richest 20% for the population (results not reported here).

**Table 3:** Odds ratios for catastrophic or impoverishing spending for those with NCDs and Injuries compared to those with Communicable Diseases, India, 2004

Variable	Catastrophic spending*	Impoverishing spending**
**Cardiovascular disease**	1.12 (0.99,1.27)	1.37 (1.23,1.53)
**Injuries**	1.22 (1.09,1.37)	1.22 (1.13,1.31)
**Cancer**	2.70 (2.10,3.10)	2.33 (1.86,2.91)
**Other NCDs**	1.12 (1.04, 1.27)	1.19 (1.09,1.24)

*Note*: 95% confidence intervals are in parentheses. Variables included in logit regression were age, sex, education, urban/rural residence *40% of annual household expenditure *less *poverty line level of spending; **Total hospitalization spending was subtracted from total household spending to assess whether a household fell below poverty line due to health spending.

## Discussion

This is the first nationally representative study in India on health spending associated with NCDs, the way such spending is financed, and the implications this spending has on catastrophic spending and impoverishment. We found that out of pocket expenses for treating NCDs rose sharply over the period from 1995-96 to 2004. The survey data we use suggest that about 40% to 50% of these expenditures are financed by household borrowing and sales of assets. These patterns indicate significant financial vulnerability to NCDs and we find both catastrophic spending and impoverishment more likely for those households that have a member hospitalized with an NCD compared to someone hospitalized with a communicable condition.

A substantial increase in utilization of health services occurred from 1995-96 and 2004 for all NCD categories. The reasons for this rise are beyond the scope of this study. However, possible reasons for this pattern include increases in prevalence, diagnosed disease, awareness and demand among patients for services, awareness and provision of services by providers, access to treatments and sharp rises in incomes. We can expect that this pattern will continue in the future.

Our data also confirm the important role that the private sector currently plays in the provision of health services for both hospital stays and outpatient visits associated with NCDs. Importantly, with its use, the financial risk is higher as out of pocket expenses per hospital stay and per outpatient visit are substantially higher in private than in the public facilities as indicated in Figures 2 and 3.

We highlight that a substantial proportion of expenditures are for medications, diagnostics and medical appliances. Medications play a critical role in reducing the risk of developing complication and diagnostic investigations are needed to determine the treatment plan and make the best use of medications. If these are foregone, physician consultation and assessment efforts will result in limited benefit.

A higher share of household expenditure is accounted for by out of pocket expenses among the richer subpopulation which seems to counter the idea that NCDs are creating a financial burden on the poor [16,17]. However, as noted already, individuals belonging to the lowest expenditure quintiles live much closer to the survival threshold, so allocating even small proportions of their low incomes will increase their likelihood of falling below the poverty line. Other monetary indicators of the financial burden suffered by households with persons with NCDs, such as income losses or premature mortality, which are not part of this study, may also contribute to this pattern.

Another way to examine the extent to which households are financially vulnerable to NCDs is to assess how expensive the costs of hospital stays are for NCDs relative to annual income (or total consumption spending). In 2004, India's income per capita was INR 25,320 while a single hospital stay for cancer or heart disease obtained from private facilities would account for anywhere between 80% and 90% of this income. Even if health care was sought from public facilities, the out of pocket expenses would still have amounted to between 40% and 50% of per capita income. A previous study has shown that the bite out of income per capita taken out by a single hospital stay increased sharply between 1995-96 and 2004 for the poorest individuals [17].

There are limitations to our study. Surveys with self-reported diseases and conditions are likely to underestimate the prevalence of different types of health conditions, and there may be a misclassification of diseases as well. We found our data's derived diabetes prevalence rates to be lower than obtained by a large diabetes survey in India with 18,000 participants which measured prevalence directly with laboratory examinations [18]. Compared to this diabetes survey, our NSSO data had lower diabetes rates in both the urban and rural areas (urban 5.9% versus 2.1%; rural 2.7% versus 0.7%) although urban-rural prevalence ratios are similar. While this may influence estimates of aggregate population-based income losses, if households accurately report all of their health spending, our results would capture the financial implications of specific health conditions at the household level, even if the overall prevalence levels are downwardly biased. Because the survey data we used provided information on outpatient healthcare use conditional only on reporting an ailment, it is possible that both healthcare use and out of pocket expenditures on outpatient care are underreported, although one recent study suggests that the impact of any underreporting is small [15].

Modeling the poverty impact for catastrophic and impoverishment also requires the assumption that there are no economies of scale in household spending [13]. Another key related assumption is that household expenditures would have remained unchanged in absence of health expenditures associated with NCDs. In the absence of additional data, the precise impact of these assumptions on our conclusions is difficult to ascertain. If household consumption were lower in the absence of health spending, for instance if increased health care expense is financed by borrowing or drawing down on savings, we may overestimate the impoverishing effects of ill health. Overestimation of poverty and catastrophic expenditure effects may also result from our reliance on data from household health care use and expenditure surveys that tend to under-estimate overall household spending, thus lowering measures of household ability to pay. On the flip side, our data do not capture the impoverishing impact of frequent expenditures for outpatient care characteristic of the chronic nature of many NCDs given that for individual households we only have information on outpatient care in the 15 days preceding the survey. Thus, our findings relating to the impoverishing impact of NCDs are subject to these appropriate caveats.

## Conclusions

This study has important implications. First, when NCDs are present, the financial risks to which Indians households are exposed are significant and result in both catastrophic spending and impoverishment. Thus, country development efforts targeted at poverty reduction must consider the impact NCDs can have on households. Despite recent significant efforts to provide risk coverage by publicly funded health insurance programs in India, social and private insurance continues to be limited and de facto financial risk coverage is provided by governments at the central and state levels in the form of subsidized public health facilities. While the use of NCD health services from public facilities results in lower out of pocket household expenses than the use of private services, they are still substantial for hospitalization, outpatient care, medications, and diagnostics. Any financing strategy should emphasize these key elements of NCDs care.

Second, the use of the private sector, at a higher out of pocket expense, implies that public sector provision may not be readily accessible or may lack capacity to care for NCDs. Understanding the reasons for this utilization pattern can help with policy solutions and should be considered in the context of overall health services financing. In addition, an understanding of the quality and efficiency of care delivered in both the public and private systems is needed to assure the financing will obtain good outputs for the money spent.

Finally, the change in utilization and out of pocket expenses from 1995-96 to 2004 and the emergence of NCDs as a leading health issue in India suggests that the need for services will increase in the future. With health services likely to serve as a key factor in controlling the economic burden of NCDs, assuring affordable access to them in the future will increasingly acquire policy prominence.

## Competing interests

The authors declare that they have no competing interests.

## Authors' contributions

MME and AM developed the conception and design of the study, analyses and interpretation of the data, AK made a substantial contribution to acquiring and analyzing the data; MME, AK, and AM have been involved in drafting and revising the manuscript; MME, AK, and AM have give final approval of the version to be published.

### Role of the funding source

This study was supported in whole by the Department for International Development (DFID), United Kingdom, through a Trust Fund to the World Bank supporting health studies in India. Anup Karan was supported by the PHFI-Wellcome Trust-UKC under the Future Faculty Programme. Neither DFID, nor PHFI or the Wellcome Trust had any role in the study design, data collection, data analysis, interpretation, or write up.

## Supplementary Material

Additional file 1 **Methodology for determining catastrophic spending and impoverishment due to out of pocket medical expenses**. Click here for file
