# Supplementary material for: The Economic impact of Non-communicable Diseases on households in India
Source: Global Health. 2012 Apr 25;8:9. doi: 10.1186/1744-8603-8-9 (PMC3383461; doi:10.1186/1744-8603-8-9)
Supplement: Additional file 1 — Methodology for determining catastrophic spending and impoverishment due to out of pocket medical expenses. [file 1744-8603-8-9-S1.DOC]

# Additional File 1

# Methodology for determining catastrophic spending and impoverishment due to out of pocket medical expenses

*Catastrophic Spending*

Survival income was defined as the poverty line level of expenditure *multiplied* by household size. Mathematically, for each household “j”, we defined a variable$D_{j}$ as

$$D_{j}=\frac{h_{j}}{E_{j}-n_{j}P}$$

Here,$h_{j}$is the combined health spending on all hospitalizations for household “j”,$E_{j}$is total household consumption spending, $n_{j}$is the size of household “j”, and P is the poverty line level of spending. Catastrophic spending is said to occur whenever$D_{j}$exceeds 0.3. We assessed the contribution of NCDs to catastrophic spending of households by estimating the following equation in a logit model:

$$C_{j}=\alpha+\sum_{i=1}^{K} \beta_{i}NCD_{ij}+\theta_{j}X_{j}+\varepsilon_{j}$$

Here$C_{j}$is a dummy variable indicating whether the household incurred catastrophic spending,$NCD_{ij}$is a dummy variable indicating the presence of major NCDs of type “i” in household “j” and$X_{j}$refers to other characteristics of household “j”.

*Impoverishment*

Household “j” and a variable indicating total household spending$E_{j}$where$E^{b}_{j}=\frac{E_{j}}{n_{j}}$indicates the household’s per capita spending (gross of health spending). Let$E^{a}_{j}=\frac{E_{j}-h_{j}}{n_{j}}$as per capita household spending after deducting total hospitalization spending.

If$E^{b}_{j}>P$and if$E^{a}_{j}<P$, we say that the health expenses of this household are impoverishing. We assessed the contribution of NCDs to catastrophic spending of households by estimating the following equation in a logit model:

$$I_{j}=\alpha+\sum_{i=1}^{K} \beta_{i}NCD_{ij}+\theta_{j}X_{j}+\varepsilon_{j}$$

Here$I_{j}$is a dummy variable indicating whether health care resulted in household impoverishment,$NCD_{ij}$is a dummy variable indicating the presence of major NCDs of type “i” in household “j” and$X_{j}$refers to other household characteristics used as controls.
